# Systematic Review of the Effects of Asbestos Exposure on the Risk of Cancer between Children and Adults

**DOI:** 10.1186/2052-4374-25-10

**Published:** 2013-07-08

**Authors:** Dongmug Kang, Min-Seung Myung, Young-Ki Kim, Jong-Eun Kim

**Affiliations:** 1 Department of Preventive and Occupational Medicine, Pusan National University School of Medicine, Yangsan, Korea; 2 Department of Occupational and Environmental Medicine, Pusan National University Yangsan Hospital, Yangsan, Korea; 3 Environmental Health Center for Asbestos, Pusan National University Yangsan Hospital, Yangsan, Korea; 4 Pusan National University School of Medicine, Yangsan, Korea

**Keywords:** Asbestos, Mesothelioma, Lung neoplasm, Childhood, Systematic review

## Abstract

Children are considerably more susceptible to enviro006Emental hazards than adults. This study was conducted to investigate whether the first asbestos exposure in childhood increases the risk of asbestos-related cancer including mesothelioma and lung cancer. MEDLINE (PubMed), Embase, and Google Scholar were searched to find relevant studies published up to July 2012. Six studies reported the relationship between age, including age during childhood, at the first asbestos exposure and mesothelioma. Among them, 4 indicated that people exposed to asbestos in childhood have a higher risk of mesothelioma than those exposed in adulthood. Meanwhile, the other 2 studies showed that asbestos exposure later in life increases the risk of mesothelioma. The results of the 2 studies including non-occupational early childhood exposure report conflicting results. There were 3 studies regarding the relationship between age at first asbestos exposure and lung cancer. However, none of them reported an association between age at first asbestos exposure and the risk of lung cancer. All studies have limitations including small numbers of subjects, the validity of the standardized mortality ratio, and different age categories at first asbestos exposure. There are only a few studies on the harmful effects of asbestos in children in the literature. Therefore, the effect of asbestos exposure during childhood remains unclear and requires further study.

## Review

### Introduction

The WHO Task Force for the Protection of Children’s Environmental Health declared, “children are not little adults” in their Bangkok statement [1]. This accounts for the fact that children are exceptionally vulnerable to both the acute and chronic effects of environmental hazards and disproportionately susceptible compared to adults [2,3]. In particular, the relationship between vulnerability during childhood and cancer risk is divided into 2 categories: a child’s susceptibility to cancer in childhood and their increased susceptibility to cancer later in life after childhood exposure. Examples of the former include ionizing radiation and diethylstilbestrol. Childhood exposure to ionizing radiation increases the risks of leukemia and other childhood cancers [4,5]. Furthermore, the use of diethylstilbestrol by mothers increases the risk of clear-cell adenocarcinoma of the vagina in their daughters during their adolescence or adulthood [1]. There may be increased susceptibility to cancer later in life after hepatitis B virus infection, consumption of salted fish, and exposure to ultraviolet (UV) light during childhood. Hepatitis B virus infection occurring earlier in life increases the risk of hepatocellular carcinoma [6]. Exposure to salted fish in the first years of life increases nasopharyngeal cancer risk in Southeast Asians [7]. Children born in Australia, where UV light density is high, or those who migrate there in the first decade of life have a higher risk of skin cancer than those who migrate at least 10 years after birth [8]. Thus, the cancer risk of asbestos exposure in childhood needs to be clarified.

Asbestos has been declared a proven human carcinogen by the US Environmental Protection Agency (EPA) and the International Agency for Research on Cancer (IARC) [9,10]. Asbestos exposure can result in numerous types of cancer. However, the 2 most common cancers related to asbestos are mesothelioma and lung cancer. Previous studies demonstrate that asbestos exposure is a well-established risk factor for malignant mesothelioma, a relatively rare tumor located mostly in the pleura [11]. Furthermore, other studies indicate that the risk of lung cancer due to asbestos is related to the duration of exposure and cumulative dose [12-16]. The carcinogenicity of asbestos has resulted in its prohibition or regulation in most countries. However, human beings are still threatened by direct or potential asbestos exposure. For instance, asbestos is still used in many places worldwide, particularly developing countries, which frequently have inadequate work safety regulations [11]. Furthermore, most countries currently experiencing or that have recently experienced rapid growth have used huge amounts of asbestos for paving roads, parking areas, school playgrounds, and race courses [17]. Asbestos has also been spread on the yards of houses to suppress red dust and mud [18].

In particular, regarding asbestos exposure in childhood, the use of asbestos in schools and playgrounds is a serious concern. According to a summary statement of the American Academy of Pediatrics, the risks posed to children by asbestos in schools are great [19]. However, despite the urgent need to study the harmful effects of asbestos on children, only a few studies have been conducted. Curiously enough, these studies concluded that asbestos exposure early in life does not increase the risk of asbestos-related cancers including mesothelioma and lung cancer [20,21]. Therefore, the present study aimed at elucidating the relationships between age of first asbestos exposure and the risk and mortality of asbestos-related cancers, i.e., mesothelioma and lung cancer.

## Materials and methods

### Literature search

MEDLINE (PubMed), Embase, and Google Scholar were searched to find relevant studies published up to July 2012. The keywords used for searching were as follows: “asbestos AND (mesothelioma OR lung cancer OR pulmonary neoplasm OR asbestosis OR pleural thickening OR pleural plaque)” in MEDLINE (PubMed); “asbestos AND (mesothelioma OR lung cancer OR pulmonary neoplasm OR asbestosis OR pleural thickening OR pleural plaque)” in Embase; and “asbestos AND (mesothelioma OR lung cancer OR pulmonary neoplasm OR asbestosis OR pleural thickening OR pleural plaque)” in Google Scholar. In principle, the searches focused on human data. To extend the search for relevant studies, in addition to mesothelioma and lung cancer, all asbestos-related diseases were included in the search terms (e.g., pulmonary neoplasm, asbestosis, pleural thickening, and pleural plaque).

### Study selection

The following inclusion and exclusion criteria were used to select highly relevant studies. The inclusion criteria were as follows: age of exposure, asbestos, mesothelioma, lung cancer, pleural plaque, asbestosis, cohort study, and case–control study. The exclusion criteria were as follows: animal study; diagnostic criteria setting; mechanism; genetic change (i.e., mutation); cancer due to other minerals, following radiation exposure, or treatment (i.e., therapy), and its strategy development; assessment method development; prevention and its strategy development; analytical tool development; clinical guidelines of asbestos-related disease development; case reports; and case studies.

**Figure 1:**
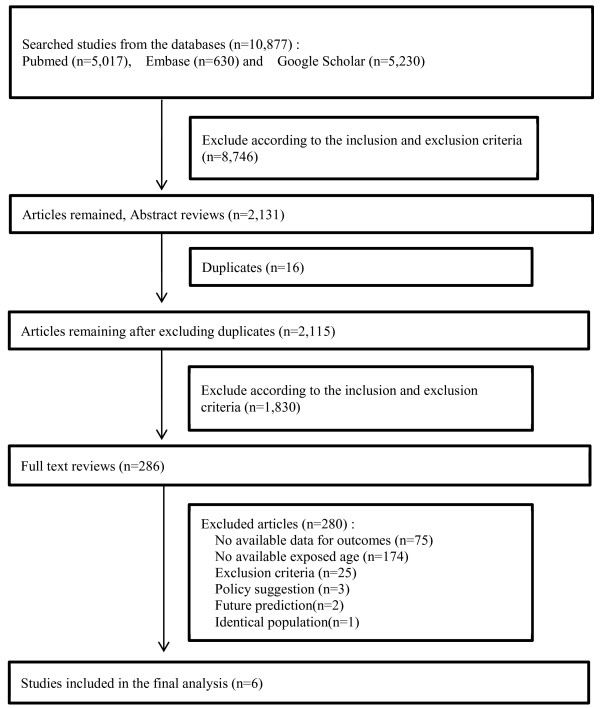
Flow diagram of data search and identification of studies.

Figure 1 shows the entire process of study selection. A total of 10,877 articles were obtained using the keywords mentioned above. The first step of study selection was title selection. According to the inclusion and exclusion criteria, 8,746 of these 10,877 articles were excluded. An additional 16 duplicate articles were excluded. Next, the abstracts of the remaining 2,115 articles were reviewed. The same selection process used for title selection was applied for abstract selection. After exclusion, 286 abstracts remained as relevant studies. The majority of excluded studies were exposure-only, animal, and clinical studies for diagnosis and/or prognosis. Full texts were subsequently reviewed to determine whether they were appropriate for the final analysis of this study. From full-text review, 6 of 286 articles, which categorized asbestos exposure age and relative risks (RRs) or odd ratios (ORs) for cancer, were determined to be relevant. The reasons why 280 of 286 studies were excluded were as follows: 75, no available data for outcomes; 174, age at exposure was unavailable; 25, clinical studies for diagnosis/treatment; 3, policy suggestion; 2, future prediction; and 1, identical population. The 6 selected studies [22-27] are shown in Table 1.

**Table 1:** Characteristics of studies included in the final analysis

**Study (reference)**		**Country**	**Period**	**Type of cancer**	**Type of exposure**	**Categorized age at first exposure**	**Asbestos exposure information**	**Confounding variables**	**Type of outcome**
1	Chen M et al., [22]	Hongkong	1981-2008	Mesothelioma and lung cancer	Occupational exposure	<20 yrs. ≥20 yrs	Age at first exposure 19.94 ± 7.92	Smoking	Standardized mortality ratios (95% CI)
							Years of asbestos exposure 31.49 ± 16.42		
2	Lacourt A et al. [23]	France	1998-2006	Mesothelioma	Occupational exposure	15 yrs, 20 yrs, 30 yrs	Age at first exposure 21.0 ± 7.0	Intensity and total duration of exposure	Odds ratio (95% CI)
							Years of asbestos exposure 27.8 ± 12.9		
3	Rake C et al. [24]	UK	2001-2006	Mesothelioma	Occupational and domestic exposure	<20 yrs, 20–29 yrs, ≥30 yrs	Categorical variables: job category, Age at first exposure, duration of employment, domestic exposure history		Odds ratio (95% CI)
4	Pira E et al. [25]	Italy	1946-2004	Mesothelioma and lung cancer	Occupational exposure	<25 yrs, 25–34 yrs, ≥35 yrs	Categorical variables: Age at first exposure, duration of employment, time since first/last employment		Standardized mortality ratios (95% CI)
5	Reid A et al. [26]	Australia	1943-2000	Mesothelioma	Residential exposure	<15 yrs, ≥15 yrs	Residential exposure (day) 1,007 ± 1,404, cumulative exposure (f/mL-yr) 5.5 ± 8	Cumulative exposure, first residence age, sex	Hazard ratio (95% CI)
6	Luce D et al. [27]	New Caledonia	1993-1995	Mesothelioma and lung cancer	Residential exposure	Birth, ≤16 yrs, >16 yrs	Categorical variables: Age at first exposure, duration of exposure	Smoking, age at first exposure, exposure duration	Odds ratio (95% CI)

### Analysis

This article was originally intended to be a meta-analysis. However, each study had different categories of age at first exposure as well as different types of outcomes including hazard ratios [26], ORs [23,24,27], and standardized mortality ratios (SMRs) [22,25]. If there were only 2 type of outcome measures (i.e., ORs and RRs), a meta-analysis could have been conducted. In rare diseases such as mesothelioma, ORs can be approximated to the RR [28]. Another problem that prevented the meta-analysis of the data was the different categories of age at first exposure to asbestos as mentioned before. Therefore, we systematically reviewed these studies instead of performing a meta-analysis.

### Results

The results of studies are divided into 2 categories according to the type of asbestos-related cancer: mesothelioma and lung cancer.

### Mesothelioma

Table 2 shows the data of the 6 studies [22-27] reporting the relationship between age at first asbestos exposure and mesothelioma. Chen [22] reported that workers aged less than 20 years in jobs involving asbestos exposure have a lower SMR of mesothelioma than those starting such jobs aged 20 years or more. Reid [26] reports similar results. The death and hazard ratios of people aged less than 15 years at first exposure were lower than that of those aged 15 years or more at first exposure. In contrast to these studies [22,26], other studies report that a younger age at first asbestos exposure results in a higher SMR or OR of mesothelioma. First, the study of Lacourt [23] showed a dramatic decrease in the OR of mesothelioma with respect to the first exposure from 15 (OR, 12.3) to 30 (OR, 1.8) years of age. This was also observed in the study by Rake [24], in which the OR decreased from 9.2 in subjects aged less than 20 years at first exposure to 1.7 in subjects aged 30 years or more at first exposure. Pira [25] and Luce [27] reported similar findings. Pira [25] reported the highest SMR in subjects aged less than 15 years (SMR, 7,968), while Luce [27] reported the highest OR at birth (OR, 52.3) among all categories of age at first exposure. Among the 6 studies, 3 studies [24,26,27] deal with non-occupational exposure to asbestos. The study by Rake [24] is on domestic exposure, while those by Reid [26] and Luce [27] are about residential (i.e., environmental) exposure. The results of these studies are inconsistent: Rake [24] and Luce [27] reported that asbestos exposure is more harmful during childhood, while Reid [26] reported contradictory results. Only 1 study [27] included birth as the age at first asbestos exposure; the corresponding OR was the highest among all age categories.

**Table 2:** The relationship between age at first asbestos exposure and mesothelioma

**Study (Type of risk)**	**Age at first exposure**	**Risk**	**95% CI**^**a**^
Chen M (2012) [22] (SMR^b^)	< 20 years	5,556.19	2,872.6-9,723.3
	≥ 20 years	7,494.63	2,428.3-17,462.5
Lacourt A (2012) [23] (OR^c^)	15 years^d^	12.3	9.0-16.9
	20 years^d^	6.5	6.1-6.9
	30 years^d^	1.8	1.1-3.0
Rake C (2009) [24] (OR^c^)	< 20 years^e^	9.2	6.4-13.1
	20-29 years^e^	3.1	1.9-5.0
	≥ 30 years^e^	1.7	0.7-3.9
Pira E (2007) [25] (SMR^b^)	< 25 years	7,968	Does not include 100
	25-34 years	4,828	Does not include 100
	≥ 35 years	2,085	Does not include 100
Reid A (2007) [26] (Hazard ratio)	< 15 years	1.00	
	≥ 15 years	3.88	2.2-6.8
Luce D 2000 [27] (OR^c^)	Birth	52.8	6.5-427
	< 20 years	20.0	1.1-368
	≥ 20 years	0.0	

^a^*CI* Confidence intervals.

^b^*SMR* Standardized Mortality Ratio.

^c^*OR* Odds Ratio.

^d^ One category of age at first exposure has different total duration of exposure which is 10, 20, 30 and 40 years. For making a comparison, 40 years of total duration of exposure was used, which shows the highest OR among those years.

^e^ One category of age at first exposure has different duration of employment which is < 5, 5–9, 10–19. ≥ 20 years and total. But for simple comparison, just data in total category of duration of employment were used. When the analysis are restricted to domestic exposure SMR among exposed male before under age 30 is 2.1 (95% CI 1.0-4.5) than age over 30, and it of exposed female before under age 30 is 1.9 (95% CI 1.1-3.2).

### Lung cancer

The data of the 3 studies [22,25,27] that reported the relationship between age at first asbestos exposure and lung cancer are shown in Table 3. According to Chen [22], subjects aged less than 20 years working in jobs involving asbestos exposure have a slightly lower SMR of lung cancer than subjects starting such work at the age of 20 years or more; workers exposed to asbestos when they were aged less than 20 years had an SMR that was 90.59% of that of workers exposed at the age of 20 years or more. Pira [25] reported that there was no association regarding the relationship between age at first asbestos exposure and lung cancer. That study reported the highest SMR of lung cancer from the age of 25 to 34 years among all categories of age at first exposure. Luce [27] presented the relationship between age at first asbestos exposure and lung cancer by sex. In the case of men, although the OR of lung cancer was highest at birth, younger age at first exposure was not a risk factor. Because the OR of men exposed before age of 20 years was lower than that of those exposed at the age of 20 years or more, the ORs of both were less than 1.0. In the case of women, the OR for first exposure at 20 years or more was the highest, which differs from the corresponding category in men. Among these 3 studies, those by Chen [22] and Pira [25] dealt with occupational exposure, while that by Luce [27] dealt with residential exposure.

**Table 3:** The relationship between age at first asbestos exposure and lung cancer

**Study (Type of risk)**	**Age at first exposure**	**Risk**	**95% CI**^**a**^
Chen M (2012) [22] (SMR^b^)	< 20 years	7.70	3.7-14.2
	≥ 20 years	8.50	2.3-21.8
Pira E (2007) [25] (SMR^b^)	< 25 years	281	Does not include 100
	25-34 years	424	Does not include 100
	≥ 35 years	269	Does not include 100
Luce D (2000) [27] (OR^c^)	Men		
	Birth	0.93^d^	0.5-1.8
	< 20 years	0.72^d^	0.2-3.1
	≥ 20 years	0.85^d^	0.3-2.9
	Women		
	Birth	2.51^d^	0.9-6.8
	< 20 years	2.03^d^	0.2-25.9
	≥ 20 years	2.93^d^	0.3-25.5

^a^*CI* Confidence intervals.

^b^*SMR* Standardized Mortality Ratio.

^c^*OR* Odds Ratio.

^d^ Odds ratio adjusted for age (≤55, 56–65, >65 years) and smoking in pack-years: four categories for men (<20, 20–39, 40–59, ≥60) and three categories for women (never smoker, <20, ≥20).

### Discussion

This article was originally intended to be a meta-analysis aimed at elucidating the relationship between childhood asbestos exposure and the risks of asbestos-related cancers. However, a literature review was performed instead due to a lack of suitable data for meta-analysis. There were 6 studies [22-27] about mesothelioma, which could be divided into 2 categories on the basis of their results: those indicating that childhood asbestos exposure is a risk factor of mesothelioma, and those suggesting that exposure later in life is a risk factor of mesothelioma. The former category includes 4 studies: Lacourt [23], Rake [24], Pira [25], and Luce [27]. The study by Lacourt [23] indicated that the risk of mesothelioma decreases with increasing age at first exposure. However, the reason for this is not explained clearly. The study merely reports that the risk of mesothelioma increases as the intensity and duration of exposure increases and that the effect of duration decreases as the age at first exposure increases [23]. Similar to Lacourt [23], Rake [24] did not provide a satisfactory explanation of the results, because that study basically aimed at providing a primary overview of the distribution of mesothelioma risk with respect to asbestos exposure in British people aged less than 30 years. However, Rake’s study [24] is that it included both occupational and residential exposure, while that of Lacourt [23] only included occupational exposure. Pira [25] reported that older age at first exposure was associated with a lower risk of mesothelioma; the author provides a reasonable explanation for this, which includes the latency effect of exposure. In another part of that study, when collected data were stratified according to age at first exposure, direct trends with respect to latency were observed. Therefore, Pira [25] concluded that the lower risk in subjects older at the time of first exposure is attributable to a shorter latency. However, that study has a limitation with respect to the validity of SMR as a risk indicator [29]. Only SMR with indirect standardization can be used because of the relatively small number of mesothelioma deaths included in that study. Direct standardization is generally preferable [30]. The data collected by Luce [27] have lower reliability, because most cases involved exposure to asbestos since birth and no patients had their first exposure after 16 years of age. Furthermore, the age at first exposure and duration of exposure are closely associated; they are also associated with time since first exposure. Therefore, the effect of age at first exposure cannot be estimated independently. The studies by Chen [22] and Reid [26] indicated that asbestos exposure later in life is a risk factor of mesothelioma. Chen [22] reported that the risk of mesothelioma increased with increasing age at first exposure. However, that study also has a limitation regarding the validity of SMR as a risk indicator. Furthermore, the study by Chen [22] was limited by the use of years of exposure, which may not adequately represent lifetime exposure, as the exposure intensity may fluctuate. The study by Reid [26] is the most reliable among all studies in the present review. The study population included people living in Wittenoom, Western Australia from 1943 to 1966. According to Reid [26], children aged less than 15 years who lived in Wittenoom had a lower rate of mesothelioma than those aged 15 years or more. These 2 groups had similar mean residence times in Wittenoom, cumulative exposure, and follow-up durations. The RR of subjects exposed at older ages was 2.4 [26]. The mesothelioma rate in those first exposed as children aged less than 15 years was approximately 40% of that of those first exposed at older ages and approximately 25% after adjusting for exposure and sex [26]. The results of these 2 studies [22,26] are corroborated by the results of an animal study [31]. That study [31] reported that the incidence rate of mesothelioma is 4-fold higher in rats inoculated intrapleurally with asbestos at 10 months of age than in rats inoculated at 2 months of age. Although asbestos exerted its effect soon after injection, the size of the effect was dependent on age. The authors [31] suggested that the lower risk in the younger rats could be due to a more efficient defense mechanism. This hypothesis could also explain the reduced risk of mesothelioma in children exposed to asbestos at a younger age.

There are relatively fewer studies about lung cancer [22,25,27] than mesothelioma in the literature. Chen [22] reported a relatively lower SMR of lung cancer in subjects exposed before 20 years of age than in those exposed at the age of 20 years or more. However, the relative ratio of the SMR of lung cancer in older age has not statistical significance. The drawbacks of that study are the same as those in the case of mesothelioma mentioned above, i.e., the use of SMRs and years of exposure. The study by Pira [25] does not demonstrate a clear relationship between lung cancer and age at first exposure to asbestos. The SMR of lung cancer is highest in people first exposed from 25 to 34 years of age. However, there are other factors that could influence the risk of lung cancer, such as smoking [32] and insoluble particles [33]. However, these studies also have limitations, because of the use of SMR as a risk indicator [29,30]. Luce [27] reported that the risk of lung cancer is related to age at first exposure and sex. This finding merely shows that the highest OR at birth, among the categories of age at first exposure, was observed in men. On the other hand, the highest OR in women was observed when first exposure was after 15 years of age. This also suggests that the risk of lung cancer due to asbestos exposure is higher in women than in men. The drawback of that study is its reliability. The data have wide confidence intervals, which indicates poor precision; [34] furthermore, the confidence intervals also include 1.0 in their ranges, which indicates a lack of statistical significance. Collectively, these 3 studies [22,25,27] show no association between age at first asbestos exposure and the risk of lung cancer. Therefore, further evaluation is needed, because their respective study populations are small and no studies excluded other possible risk factors in their statistical analyses.

None of the studies [22-27] in this review demonstrated a clear association between age at first asbestos exposure with a risk of mesothelioma or lung cancer. In general, there are important age-related differences in the susceptibility to environmental toxins [35-37]. Experimental and epidemiologic data indicate that infants and children have a greater risk of negative effects from a number of environmental toxins than adults because of differential exposure or physiological immaturity [38]. In the case of asbestos-related cancer, some studies [23-25,27] support this hypothesis while others do not [22,26]. In some studies [22,26], older age at first exposure to asbestos appears to be a risk factor of mesothelioma. However, those first exposed as children have many years left to live; hence, their lifetime risk may not be lower than that of people exposed when they were older.

The present study revealed several problems in previous studies that need to be overcome. First of all, there are very few studies on the relationship between age at first asbestos exposure and the risk of asbestos-related cancer despite the importance of understanding the harmful effects of asbestos on children. Although we cannot be certain that all relevant studies were included in our search results, extended databases were searched, including PubMed, Embase, and Google Scholar; these are considered to be the main databases for systematic reviews. Regardless, the fact that we only found 6 studies highlights the lack of research on this subject. The second problem to be overcome is the necessity of standardized age categories regarding age at first exposure to asbestos. The studies in this review utilized a variety of categories, but mainly subjects younger and older than 20 years (i.e., children and adults). However, vulnerability varies greatly during childhood. Therefore, a single category for children will not adequately represent all characteristics of childhood. Hence, childhood should be divided into different categories to evaluate the effect of age at first exposure. A recent helpful attempt to harmonize the terminology for stages of childhood suggests 5 age groups: preterm (in utero), term-newborn (0–27 days), infants and toddlers (28 days to 23 months), children (2–11 years), and adolescents (12 to 16–18 years depending on region) [39]. When this prerequisite is met, the susceptible age to asbestos exposure will be determined similar to those for dioxin (in nursing infants) [40], tobacco smoke (in young children) [41], polycyclic aromatic hydrocarbon (PAH; in young children) [41], and radiation (in teenage girls) [35]. The third problem to be overcome is related to the types of outcome measures. The studies reviewed in this article used hazard ratios [26], ORs [23,24,27], and SMRs [22,25]. However, SMR is not generally preferable; it is usually used when there are few deaths in a cohort. This outcome measure cannot be modified in cohort studies [22,25]. Therefore, larger cohorts obtained through multi-center studies are required to improve reliability. The final problem to be overcome is the difficulty in collecting data regarding age at first exposure to asbestos. Four studies [22-25] in this review collected data regarding occupational asbestos exposure, while the other 2 did not [26,27]. In cohorts of asbestos-exposed workers, age at first employment could be treated as the age at first exposure to asbestos. However, during childhood, most exposure to asbestos comes from the environment. Therefore, the precise age at first exposure to asbestos is difficult to determine. This is one possible reason why only a few studies on this subject have been conducted. The study by Reid [26] presents a possible solution to this problem. People who move to given area where asbestos exposure is high would be a perfect cohort for studying this subject. Unless the migrants have been exposed to asbestos before, the age at migration could be considered the age at first asbestos exposure.

## Conclusions

In conclusion, our review of studies on the relationship between age at first asbestos exposure and the risk of asbestos-related cancer cannot draw any clear conclusions due to the lack quantity and quality of previous studies. However, this review highlights the dearth of studies on this subject as well as the problems that future studies need to overcome.

## Competing interests

The authors declare that they have no competing interests.

## Authors’ contribution

DK carried out analysis and writing. MM conducted literature searching and writing. YK carried out review of articles. JK conducted qualitative review. All authors read and approved the final manuscript.
